# Modelling the maternal‐fetal interface: An in vitro approach to investigate nutrient and drug transport across the human placenta

**DOI:** 10.1111/jcmm.70151

**Published:** 2024-10-18

**Authors:** Barbara Fuenzalida, Virginia Basler, Nadja Koechli, Nan Yi, Frantisek Staud, Christiane Albrecht

**Affiliations:** ^1^ Institute of Biochemistry and Molecular Medicine, Faculty of Medicine University of Bern Bern Switzerland; ^2^ Department of Pharmacology and Toxicology, Faculty of Pharmacy in Hradec Kralove Charles University Hradec Kralove Czech Republic

**Keywords:** co‐culture, endothelial cell, LAT1, P‐gp, placental barrier, polarized monolayer, primary trophoblast, transport

## Abstract

The placenta plays a critical role in maternal‐fetal nutrient transport and fetal protection against drugs. Creating physiological in vitro models to study these processes is crucial, but technically challenging. This study introduces an efficient cell model that mimics the human placental barrier using co‐cultures of primary trophoblasts and primary human umbilical vein endothelial cells (HUVEC) on a Transwell^®^‐based system. Monolayer formation was examined over 7 days by determining transepithelial electrical resistance (TEER), permeability of Lucifer yellow (LY) and inulin, localization of transport proteins at the trophoblast membrane (immunofluorescence), and syncytialization markers (RT‐qPCR/ELISA). We analysed diffusion‐based (caffeine/antipyrine) and transport‐based (leucine/Rhodamine‐123) processes to study the transfer of physiologically relevant compounds. The latter relies on the adequate localization and function of the amino‐acid transporter LAT1 and the drug transporter P‐glycoprotein (P‐gp) which were studied by immunofluorescence microscopy and application of respective inhibitors (2‐Amino‐2‐norbornanecarboxylic acid (BCH) for LAT1; cyclosporine‐A for P‐gp). The formation of functional monolayer(s) was confirmed by increasing TEER values, low LY transfer rates, minimal inulin leakage, and appropriate expression/release of syncytialization markers. These results were supported by microscopic monitoring of monolayer formation. LAT1 was identified on the apical and basal sides of the trophoblast monolayer, while P‐gp was apically localized. Transport assays confirmed the inhibition of LAT1 by BCH, reducing both intracellular leucine levels and leucine transport to the basal compartment. Inhibiting P‐gp with cyclosporine‐A increased intracellular Rhodamine‐123 concentrations. Our in vitro model mimics key aspects of the human placental barrier. It represents a powerful tool to study nutrient and drug transport mechanisms across the placenta, assisting in evaluating safer pregnancy therapies.

## 1 INTRODUCTION

The human placenta is a vital, highly specialized organ that plays a pivotal role as an interface between the maternal and fetal environment, facilitating essential exchanges in nutrition, waste removal and respiration for the developing fetus.^1^, ^2^ The placental barrier consists primarily of trophoblast cells, which include syncytiotrophoblast (STB) and cytotrophoblast cells (CTB), and an endothelial layer.^3^ The STB layer, formed by the fusion and polarization of CTB, is responsible for mediating the transfer of nutrients and various molecules between the maternal and fetal circulation. These cells feature an apical membrane in contact with maternal blood and a basal membrane facing towards placental endothelial cells.^3^, ^4^ This intricate structure, the placental barrier, serves as a protective shield, safeguarding the developing fetus from exposure to xenobiotics.^5^, ^6^, ^7^, ^8^ However, it is important to acknowledge that certain drugs can traverse the placental barrier,^5^, ^7^ potentially influencing fetal development.^9^ Despite the lack of comprehensive studies on drug safety during pregnancy, the use of medications in pregnant women is widespread.^10^, ^11^ However, investigating drug transport directly in humans is ethically questionable and technically difficult. Therefore, there is an urgent need for novel experimental models that closely replicate the maternal‐fetal interface to assess the kinetics and toxicity of existing and emerging drugs during pregnancy.

It is crucial to note that significant disparities in placentation exist between humans and animals,^12^, ^13^ making animal models less suitable for experimental research. In alignment with this, the FDA has recommended a reduction in animal use in research and is prioritizing the 3R principle of replacement as of the end of 2022.^14^ Other recently established cellular models, such as co‐culturing immortalized trophoblast and placental microvascular endothelial cells,^15^ have limitations due to the potential influence of cell line immortalization or malignancy on their susceptibility to toxic agents.^16^, ^17^ Certain drug transporters may also not be fully expressed in these cell lines.^18^ Furthermore, although placenta‐on‐a‐chip devices have been developed to emulate the metabolic processes and active transport within the human placenta, they have the drawback of absorbing small hydrophilic molecules, which can hinder reliable drug transfer studies.^19^ Existing physiological ex vivo and in vitro models are also suboptimal for routine drug transfer investigations. For instance, the ex vivo dual perfusion system of the human placenta, while effective, is costly, technically demanding, and harbours experimental limitations.^20^ In our previous work, we developed a confluent monolayer using primary trophoblast cells from the human placenta on a Transwell^®^ system.^21^ However, this model lacks physiologically important components, notably the contribution of fetal endothelial cells.

In response to these challenges, we have established a novel co‐culture model employing primary trophoblast cells and primary human umbilical vein endothelial cells (HUVEC) on a pre‐coated polycarbonate Transwell^®^ system, effectively simulating the maternal‐fetal interface. To validate our model, we conducted a comprehensive evaluation, including (1) characterizing the electrophysical properties and permeability of the co‐culture, (2) performing immunostaining for epithelial and endothelial markers during monolayer formation, (3) assessing the expression of syncytial markers and secretion of pregnancy‐related hormones, (4) evaluating the polarization of the trophoblast cells using membrane markers, (5) conducting passive diffusion experiments in the co‐culture model and (6) determining the functionality of the model through the assessment of active transport processes facilitated by the L‐ type amino acid transporter 1 (LAT1) and the ATP‐binding cassette (ABC) drug transporter P‐glycoprotein (P‐gp).

## 2 MATERIALS AND METHODS

### 2.1 Obtaining biological specimens

Human placentas were collected from uncomplicated pregnancies following elective Caesarean section beyond 37 weeks of gestation at the Lindenhofgruppe, Bern, Switzerland. The study adhered to the ethical principles outlined in the Declaration of Helsinki and received approval from the Canton of Bern, Switzerland (Basec Nr. 2016‐00250). Informed consent was obtained from patients, along with their clinical data in coded form.

### 2.2 Isolation of primary cytotrophoblasts

Primary trophoblast cells were isolated from freshly obtained healthy‐term placental tissues. Villous cytotrophoblast cells were specifically extracted from term placental tissues through a well‐established enzymatic digestion procedure using 0.25% trypsin (Thermo Fisher Scientific, USA) and 300 IU/mL DNAse (Sigma Aldrich, USA), in combination with the Percoll^®^ (Sigma Aldrich, USA) gradient separation method, as previously detailed.^22^, ^23^

The purity of the trophoblast cell population was rigorously assessed using specific cellular markers, including anti‐cytokeratin 7 (CK7) for epithelial identification and anti‐vimentin for mesenchymal cell characterization (Novus Biologicals, USA). Flow cytometry analysis was conducted using a BD FACSDiva flow cytometer (LSRII, BD Biosciences, USA). The results indicated that the isolated trophoblasts exhibited a high degree of purity, ranging from 93% to 98%.

### 2.3 Isolation of primary endothelial cells

HUVEC were isolated from fresh‐term umbilical cords. A 10% solution of Collagenase Type I (Thermo Fisher Scientific, USA), prepared in endothelial cell medium (ECM; Curio Biotech SA, Switzerland), was used to perfuse the vein. Following a 20min incubation at 37°C,^24^ the cell suspension was centrifuged at 1000 RCF for 10 min. The resulting cellular pellet was resuspended in ECM supplemented with 5% fetal bovine serum (FBS). This suspension was subsequently plated into a T25 cm^2^ flask that had been previously coated with 1% gelatin (Sigma Aldrich, USA). The cells were placed in an incubator maintained at 37°C and 5% CO_2_ for cell culture. All experiments were performed with HUVEC grown between passages 3 and 5.

### 2.4 Co‐culture monolayer formation

Transwell^®^ inserts with 0.4 μM pore polyester (PET) membrane (Corning Incorporated, USA) were coated with Matrigel (Corning Incorporated, USA; 25 μg/cm^2^) for 30 min at 37°C. HUVEC were seeded at a density of 0.5 cells/cm^2^ onto the basal membrane of the Transwell^®^ insert and were placed into the incubator for approximately 3 h until cells were attached. After attachment of the HUVEC, primary trophoblasts were plated at a density of 1.2 cells/cm^2^ onto the apical membrane of the Transwell^®^ insert to obtain a co‐culture.^21^ The co‐cultured cells were placed in a mixture of Dulbecco's Modified Eagle Medium‐ high glucose (DMEM‐HG, Thermo Fisher Scientific, USA) and ECM medium (Curio Biotech SA, Switzerland) at a ratio of 70:30 (DMEM‐ECM), supplemented with 5% FBS and 1× antibiotic‐antimycotic (Thermo Fisher Scientific, USA). Co‐cultures were grown in a humidified incubator under 5% CO_2_ atmosphere at 37°C.

The transepithelial/transendothelial electrical resistance (TEER) and the capacitance (Ccl) were measured continuously and automatically using the cellZscope^®^ device (nanoAnalytics, Germany). 1.5 mL of DMEM‐ECM + FBS 5% was added to each well (basal compartment) and 0.8 mL to each insert (apical compartment).

### 2.5 Apparent permeability coefficient measurements

To determine the apparent permeability coefficient (Papp), rates of passive transfer of the low molecular weight substance Lucifer yellow (LY; 457 Da) across the cell monolayer were determined. The Transwells^®^ were transferred to a 12‐well plate, adding cell‐free Transwells^®^ as controls, and were equilibrated in phenol red‐free DMEM‐LG medium for 30 min. A LY standard curve ranging from 0.05 to 50 μM was prepared using a 25 mM stock solution. Then 50 μM LY dilution in medium was added into the donor chamber at 37°C for 2 h. 100 μL aliquots were collected from the receiver chamber every 30 min. The fluorescence was measured at Ex/Em = 450/530 nm using a Flex Station II fluorescence microplate reader.^21^ Papp was calculated using the equation Papp (cm/s) = (dQ/dt)/(A × C_0_), where dQ/dt is the rate of the substrate appearance on the receiver chamber (μmol/s); A represents the surface area of the filter membrane (cm^2^) and C_0_ the initial concentration of the substrate on the donor chamber (μmol/mL).

### 2.6 mRNA isolation and quantitative RT‐PCR

According to the manufacturer's protocol, total RNA was extracted from the trophoblasts in the Transwell^®^ using TRI reagent (Invitrogen, UK). Total RNA (0.5 μg) was reverse transcribed to cDNA using the GoScript™ Reverse Transcription System (Promega, USA). Six different syncytialization markers were analysed: syncytin 1, syncytin 2, human chorionic gonadotropin (hCG), dysferlin, human placental growth factor (hPlGF) and human placental lactogen (hPL). Quantitative reverse transcription‐PCR (qRT‐PCR) was performed, as previously described.^25^ qRT‐PCR was carried out on the CFX qRT‐PCR system using SYBR^®^ Green PCR master mix detection kit (Promega, USA). The primer sequences are listed in Table 1. Tyrosine 3‐monooxygenase/tryptophan 5‐monooxygenase (YWHAZ) was used as a reference gene. The relative gene expression was calculated using the 2^−ΔΔCq^ method.

**TABLE 1:** Nucleotide sequences of primers.

Gene	Primer forward (5′‐3′)
hCG	F‐CGGGACATGGGCATCCAA
	R‐ GCGCACATCGCGGTAGTT
Syncytin 1	F‐GATATTTGGCTAAGGAGGTGATGTC
	R‐ GAAGGCCCTTCATAACCAATGA
Syncytin 2	F‐GCTGTCCCTGGTGTTTCAGT
	R‐ CCTTCACTAGCAGCCTACCG
hPL	F‐GCTATGCTCCAAGCCCAT
	R‐TGCAGGAATGAATACTTCTGGT
hPlGF	F‐CTCCTAAAGATCCGTTCTG
	R‐CTTTCCGGCTTCATCTTC
Dysferlin	F‐CCGTATTTGGAAAGATGTT
	R‐ AGGAGGTCATAGTCATAGA
YWHAZ	F‐CCGTTACTTGGCTGAGGTTG
	R‐AGTTAAGGGCCAGACCCAGT

### 2.7 Measurement of protein secretion by ELISA

The secretion of β‐hCG into the trophoblast media after 1–7 days of growth on Transwell^®^ inserts was measured using a human hCG (intact) ELISA kit (Sigma Aldrich, USA), following the manufacturer's instructions. The absorbance was measured at a wavelength of 450 nm on a Vmax microplate reader. The hCG concentrations from the trophoblast supernatants were interpolated from the standard curve. The hPL ELISA was conducted using an in‐house established ELISA. A 96‐well microplate was coated with 100 μL of polyclonal rabbit anti‐hPL (1:500; 4°C; 18 h; Dako, Denmark). On the next day, the plate was blocked in 250 μL of blocking buffer (0.5% bovine serum albumin (BSA) in Phosphate Buffered Saline (PBS); 2 h; room temperature (RT)). Thereafter, 100 μL of the media samples and a standard curve (0–400 ng/mL) were added to the plate and incubated for 90 min at 37°C. After washing three times with 250 μL of PBS plus 0.1% Tween‐20 (PBS‐T20), peroxidase‐conjugated rabbit anti‐hPL was added (1:1000; Dako, Denmark) and samples were incubated for 1 h at 37°C. After four washing steps with 250 μL of PBS‐T20, the substrate buffer (o‐Phenylenediamine dihydrochloride; 1 mg/mL) was added to the plate and incubated at RT in the dark for 30 min. The reaction was stopped with 100 μL of H_2_SO_4_ (2.5 M), and the absorbance was measured at 492 nM.

### 2.8 Immunofluorescence analysis

The membranes of the Transwell^®^ inserts including trophoblasts and HUVEC were formalin‐fixed (4% buffered formalin solution, 20 min, RT, Thermo Fisher Scientific, USA) and stained for immunofluorescence microscopy. Briefly, the membranes harbouring either only trophoblasts, only HUVEC, or the co‐cultures were collected from day 1 to 7 and incubated (1 h, RT) in blocking buffer (100 mmol/L NaCl, 0.05% Triton‐100, 5% BSA 50 mmol/L Tris/HCl, pH 7.5). Membranes containing HUVEC were incubated for 18 h at 4°C with the primary anti‐rabbit antibody (1:50) against the cluster of differentiation 31 (CD31) (endothelial marker; Genetex, USA). Trophoblast containing membranes were incubated with the primary anti‐mouse antibody anti‐CK7 (epithelial marker; Dako, Denmark; 1:100). Membranes from the inserts containing co‐cultures at day 5 were incubated with primary anti‐mouse antibody against P‐gp (1:50), anti‐rabbit anti‐ATP‐binding cassette transporter G1 (ABCG1; 1:50) (Genetex, USA) and anti‐rabbit antiLAT1 (1:50) (Transgenic Inc., Japan). Then, cells were incubated for 1 h at RT with Alexa Fluor^®^ 488‐conjugated goat anti‐mouse (H + L, λexc/ λem: 495/568 nm, 1:1000 dilution), Alexa Fluor^®^ 568‐conjugated goat anti‐rabbit IgG (H + L, λexc/λem: 578/603 nm, 1:1000 dilution) (Thermo Fisher Scientific, USA) in blocking buffer containing 0.1 μg/mL DAPI (4′,6‐Diamidino‐2‐Phenylindole, Dihydrochloride) (Invitrogen, USA).^26^ The membranes of the Transwell^®^ inserts were inspected using a Zeiss LSM 710 Confocal microscope Airyscan (Zeiss, Germany). The images were processed with ImageJ version 2.1.0 (NIH, USA).

### 2.9 Cell viability assay

Cell viability was evaluated in trophoblasts and HUVEC using 3‐(4,5‐dimethylthiazol‐2‐yl)‐2,5‐diphenyltetrazolium bromide (MTT) (Sigma Aldrich, USA). 1 × 10^5^ cells were seeded in a 96‐multiwell plate and grown for 3 days. The effect on cell viability was tested for the following compounds at the indicated concentrations: caffeine (0.9 mM) (Sigma Aldrich, USA), antipyrine (0.43 mM) (Sigma Aldrich, USA), Rhodamine 123 (10 μM) (Medchem Express, USA), Cyclosporine A (CsA) (10 μM) (Medchem Express, USA), [^3^H]‐L‐leucine (1.5 μCi/mL) (ARC, Saint Louis, USA), leucine (150 μM) (Sigma Aldrich, USA) and 2‐Amino‐2‐norbornanecarboxylic acid (BCH; 4 mM) (Sigma Aldrich, USA). The cells were incubated with the different compounds for 1, 3, 6 and 24 h. At the indicated time points, the supernatants were removed and the cells were incubated with 20 μL of 12 mM MTT stock solution in 100 μL of medium per well for 4 h at 37°C in 5% CO_2_. The medium was removed and 150 μL of dimethyl sulfoxide (DMSO) was added. After mixing in darkness at RT for 10 min, the absorbance was measured on a microplate reader at 570 nm.

### 2.10 Diffusion experiments

The diffusion characteristics of caffeine, antipyrine and inulin through the cell layer(s) were evaluated on day 5 post seeding. Caffeine (0.9 mM), antipyrine (0.43 mM) or inulin (0.19 mM) was dissolved in FBS‐depleted, Phenol red‐free DMEM‐LG medium. The single compounds were added in individual experiments to the apical compartment of the Transwell^®^ inserts as previously described with minor modifications.^27^, ^28^ Samples from the basal and apical compartments were collected between 0 and 24 h and measured using capillary zone electrophoresis (CZE) (Beckman Coulter, USA). The assay comprises hydrodynamic injection of an alkalinized sample, and analysis at pH 9.3 in a phosphate/tetraborate CZE buffer with 50 mM SDS and 2% methanol as previously described with minor modifications^20^ including the use of creatinine (6.2 mM) as internal standard control (IST). A mixture of 100 μL sample from the apical or basal compartment of the Transwell^®^ and 40 μL of IST was loaded onto the CZE device. Analyte detection was performed at 254 nm (PDA detector). Caffeine, antipyrine and creatinine (IST) were detected at 7.9, 10.1 and 5.6 min, respectively. Quantification was performed based on a seven level internal calibration range (caffeine: 0.08–5 mM; antipyrine: 0.005–0.85 mM). Inulin was measured using a Flex Station II fluorescence microplate reader at Ex/Em = 495/519 nm.

### 2.11 Leucine transport assay

The transport assays were performed in the absence and presence of BCH (Sigma Aldrich, USA), an inhibitor of system L amino acid transporters.^29^, ^30^ The inserts at day 5 post seeding were washed with pre‐warmed Na^+^‐free Hank's buffer (125 mM choline chloride, 25 mM HEPES, 4.8 mM KCl, 1.2 mM MgSO_4_, 1.2 mM KH_2_PO_4_, 1.3 mM CaCl_2_, 5.6 mM glucose, adjusted pH to 7.4 with 2.5 M Tris–HCl), then equilibrated in Na^+^‐free Hank's buffer with/without 4 mM BCH for 30 min at 37°C in both compartments. In the upper compartment, the buffer was replaced with Na^+^‐free Hank's containing 150 μM L‐leucine including 1.5 μCi/mL [^3^H]‐labelled L‐leucine, 300 μM glutamine, with or without BCH (4 mM). 50 μL medium samples from the apical and basal compartments were collected into 3 mL Irgasafe‐2 plus scintillation liquid (Zinsser Analytic, Germany) for up to 24 h. Additionally, membranes of the Transwell^®^ inserts were collected during the experiment at 3, 8 and 24 h and were put into separate vials filled with scintillation liquid. The radioactivity was measured using a beta counter (PerkinElmer, Waltham, USA), as previously detailed with some modifications.^31^

### 2.12 P‐gp‐mediated Rhodamine 123 transport

To determine the expression and activity of the P‐gp transporter, we used the substrate Rhodamine 123 and the inhibitor CsA.

On day 5 post seeding, the inserts were washed and equilibrated for 30 min at 37°C with FBS‐depleted, Phenol red‐free DMEM‐LG medium. Briefly, the inhibitor CsA (10 μM) was incubated for 30 min at 37°C in both compartments. In the upper compartment, the medium was replaced with an FBS‐depleted, Phenol red‐free DMEM‐LG medium containing Rhodamine 123 (10 μM) with or without CsA (10 μM). 50 μL medium samples from the apical and basal compartment were collected and transferred into a 96‐well black plate with clear bottom (Corning Incorporated, USA) for 0–6 h and then measured using a Flex Station II fluorescence microplate reader at Ex/Em = 500/525 nm. At the end of the experiment, Transwell^®^ inserts were fixed with paraformaldehyde 4% and then analysed using a Zeiss LSM 710 Confocal microscope Airyscan (Zeiss, Germany). Fluorescence quantification was performed by selecting the entire membrane of the trophoblasts and correcting for both the area and background of the images. The background was determined by navigating through the z‐stack until a black background outside the Transwell^®^ insert was reached. Consistent imaging settings were applied across all samples, and representative images from three independent experiments were analysed. The images were processed with ImageJ version 2.1.0 (NIH, USA).

### 2.13 Statistical analysis

Results are presented as the mean ± S.E.M., where *n* indicates the number of separate experiments using primary trophoblasts alone, HUVEC alone, or co‐cultures on the Transwell^®^ inserts. The groups were compared using ANOVA. *p ≤ 0.05, **p ≤ 0.01, *** *p* ≤ 0.001 and ****p ≤ 0.0001 were considered statistically significant. GraphPad Prism 9.5.0 (GraphPad Software Inc., USA) was used to analyse the data and create the figures.

## 3 RESULTS

### 3.1 Monolayer formation and leakiness in the mono‐ and co‐culture cell models

**FIGURE 1:**
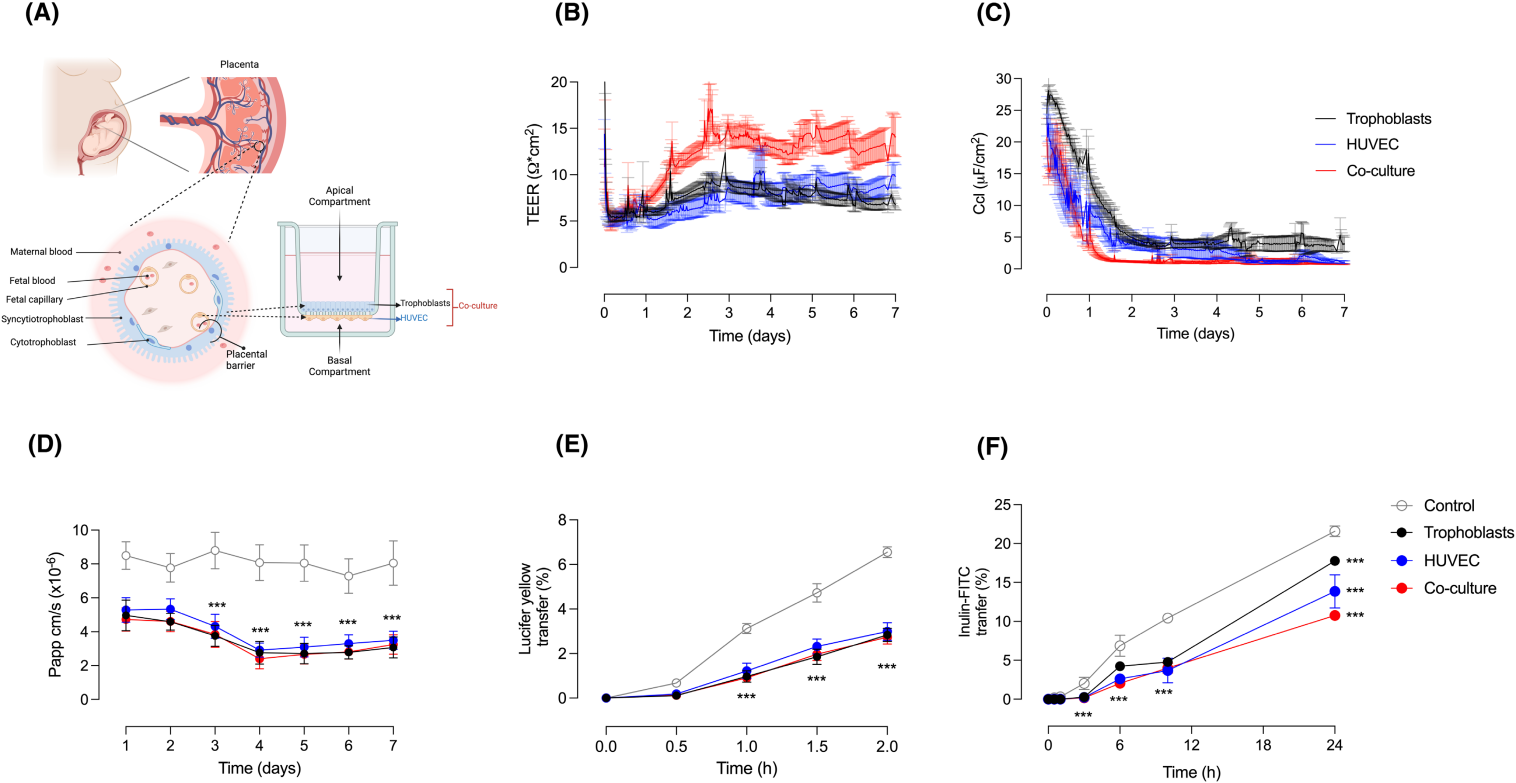
Assessment of monolayer formation and leakiness in the mono‐ and co‐culture cell models. (A) This figure provides an overview of the components of the in vitro model and illustrates how it mimics the placental barrier. A Transwell^®^ system including primary trophoblasts and primary endothelial cells grown on a microporous membrane simulates the placental interface which in vivo separates the maternal and fetal circulation. Primary trophoblast cells were seeded in the apical compartment (representing the maternal side), while endothelial cells were grown on the basal side (representing the fetal part) of the membrane. The cells in the co‐culture system were allowed to grow for 7 days, ensuring the formation of a continuous monolayer. Biochemical and biophysical assessments were conducted to confirm the cellular integrity and to determine the transcellular leakage. (B) Transepithelial/transendothelial electrical resistance (TEER, [Ω*cm^2^]) and (C) Capacitance (Ccl, [μF/cm^2^]) measurements in the cellZscope^®^ system. (D) Apparent permeability coefficient measurement (Papp, [cm/s]) was calculated by measuring the rates of passive transfer of the paracellular pathway marker Lucifer yellow (50 μM) from days 1 to 7. (E) Lucifer yellow (50 μM, 2 h) and (F) inulin‐FITC (0.19 mM, 24 h) were applied to evaluate cell leakiness across the cell monolayer(s). Results are shown for primary trophoblasts (black), human umbilical vein endothelial cells (HUVEC) (blue), co‐cultures (trophoblasts + HUVEC; red) and controls (cell‐free Transwell^®^ inserts, grey). Data are shown as the mean ± S.E.M, *n* = 6–8 per group. For comparison of trophoblast mono‐cultures, HUVEC mono‐cultures or trophoblast/HUVEC co‐cultures with controls, ANOVA was applied. ***p < 0.001 versus control. Figure 1A was created with BioRender.com.

Trophoblast cells were seeded on the apical membrane of a Transwell^®^, while HUVEC were added to the basal membrane (Figure 1A). To evaluate monolayer formation, the Transwell^®^ inserts containing trophoblasts or HUVEC in mono‐culture, as well as the co‐culture of both cell types, were monitored using the cellZscope^®^ device. The cellZscope^®^ was operated continuously for 7 days, with medium changes every 2 days. An increase in TEER was observed for all cell conditions, including trophoblasts alone, HUVEC alone, and co‐cultures of both cell types, starting from day 1. The TEER values reached a peak between day 2 and day 3 and remained stable until day 7 (Figure 1B). The TEER values in the co‐culture were higher than those in the mono‐cultures, indicating increased resistance due to the presence of both cell types. Additionally, the Ccl of all cell types decreased after day 1 and remained stable until day 7, with values consistently below 5 μF/cm^2^, indicative of monolayer formation (Figure 1C).

The Papp of LY gradually decreased from day 1 to day 4 and remained stable until day 7 for all cellular setups, that is, both for the trophoblast and HUVEC mono‐cultures as well as for the co‐culture system (Figure 1D). Notably, the permeability of LY in the control group (cell‐free Transwell^®^ inserts, open grey circles) was significantly higher compared to all cell‐containing groups (Figure 1D). To assess leakage, the transfer of LY and inulin, two differently‐sized molecules (457 Da and 5200 Da, respectively), was evaluated. After 2 h, only 3% of LY was transferred to the basal compartment in the co‐culture, compared to 7% in the control (Figure 1E). Inulin transfer to the basal compartments after 24 h was 17%, 14% and 10% in trophoblast, HUVEC and co‐culture, respectively, compared to 22% in the control (Figure 1F).

### 3.2 Monitoring of monolayer formation using confocal microscopy in Transwell^®^ inserts over 7 days

**FIGURE 2:**
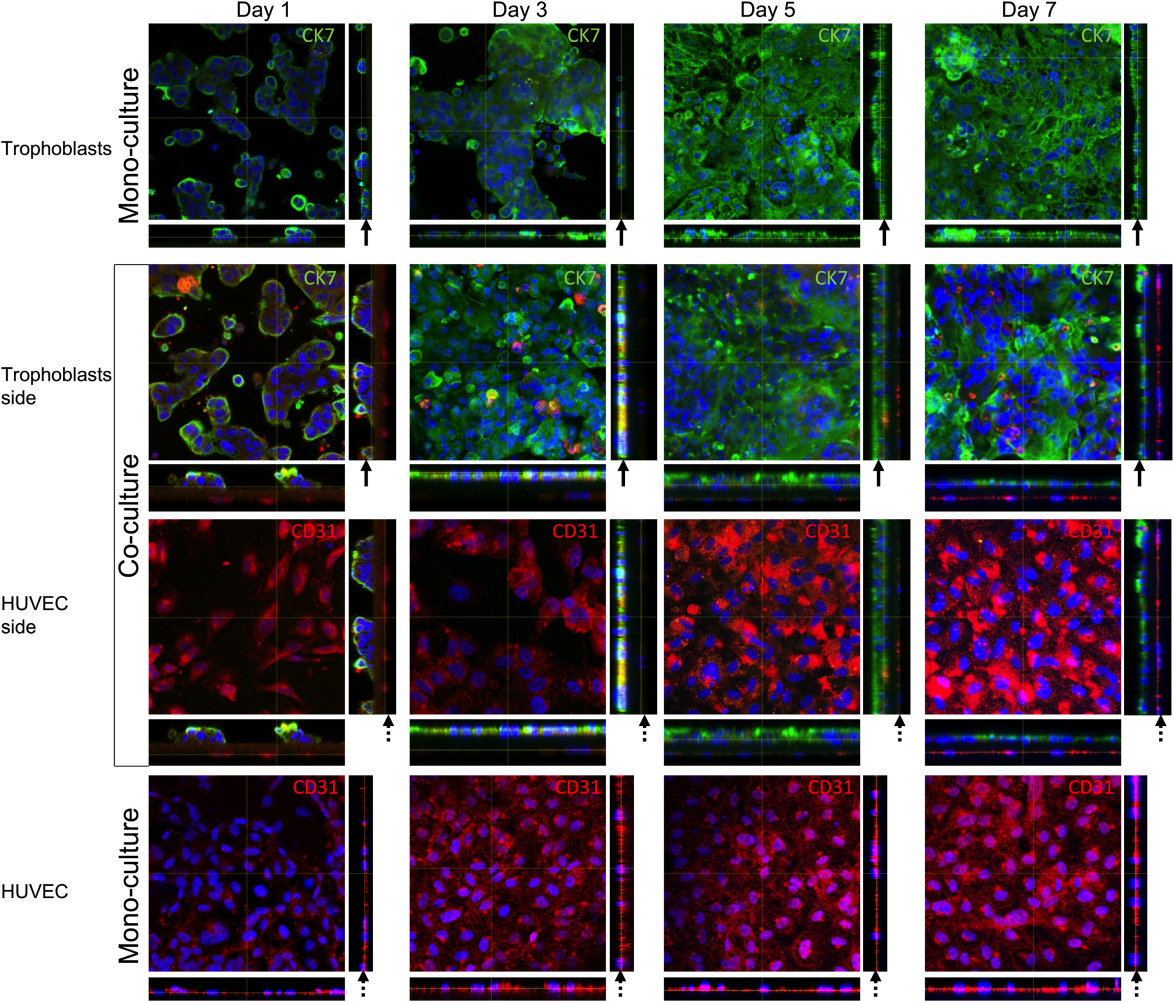
Monolayer formation in Transwell^®^ inserts between days 1 and 7. Cytokeratin 7 expression (CK7, 1:100, green) was determined by immunofluorescence in the Transwell^®^ inserts containing trophoblasts (mono‐culture) and co‐culture of trophoblasts/HUVEC. Cluster Differentiation 31 expression (CD31, 1:50, red) was analysed by indirect immunofluorescence in the Transwell^®^ inserts containing HUVEC (mono‐culture) and the co‐culture of trophoblasts/HUVEC. 4′,6‐Diamidino‐2‐Phenylindole, Dihydrochloride (DAPI) was used to stain the nuclei (0.1 μg/mL, blue). The immunofluorescence images show the merge of DAPI and the corresponding antibody. The full black arrow indicates the trophoblast part in the membrane of the Transwell^®^, while the dashed black arrow depicts the HUVEC section. Representative images of the Z‐stacks are depicted using orthogonal projections through the Z‐stack, with 40× objective in a Zeiss LSM 710 Confocal microscope Airyscan. *n* = 3 per group.

Monolayer formation was assessed from day 1 to 7 using immunofluorescence staining with the epithelial (trophoblast) marker CK7 and the endothelial marker CD31 (Figure 2), demonstrating the progressive development of a monolayer in both mono‐culture (trophoblasts and HUVEC) and co‐culture conditions. In trophoblast mono‐cultures, the formation of a monolayer was observed between days 5 and 7, showing a high confluency (Figure 2, upper panel). Orthogonal images in the z‐stack demonstrate ample space between the cells on day 1, whereas by day 5 complete coverage was evident (indicated by black arrows).

In the co‐culture, a distinct time course for monolayer formation was observed between trophoblasts and HUVEC. The trophoblast side showed monolayer formation by day 3, while the HUVEC side reached confluency by day 5 (Figure 2, middle panel). This information was also verified by evaluating the z‐stack images showing monolayer formation in the trophoblasts (black arrow) and the HUVEC (black arrows with dashed lines). In HUVEC mono‐cultures a fully formed monolayer was found by day 3, which remained stable until day 7 (Figure 2, lower panel). Again, the z‐stack projections demonstrate the confluency of the HUVEC monolayer (black arrows with dashed lines). Negative controls are shown in the supplementary information (Figure S1).

### 3.3 Trophoblast syncytialization in mono‐ and co‐cultures

To assess whether the syncytialization process of trophoblast cells was affected in the co‐culture model, we measured the mRNA expression and partly also the supernatants' protein levels of different syncytialization markers both under mono‐ and co‐culture conditions.

**FIGURE 3:**
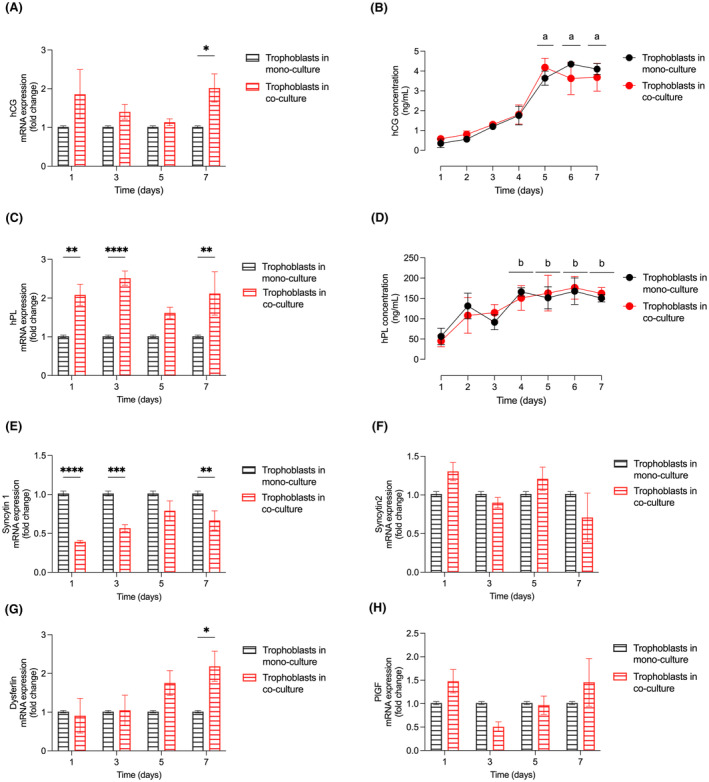
Expression of syncytial markers in trophoblast mono‐ and co‐cultures. (A) The expression of human chorionic gonadotrophin (hCG) as an indicator for syncytium formation was determined on mRNA level by qRT‐PCR. (B) Secretion of the hCG protein into the supernatant analysed by ELISA. (C) mRNA levels of human placenta lactogen (hPL), (D) Secretion of hPL protein analysed by ELISA. In addition, the mRNA expression of (E) Syncytin 1, (F) Syncytin 2, (G) Dysferlin and (H) Human placental growth factor (hPlGF) was determined by qRT‐PCR. (A, C, E–H) The mRNA levels of syncytialization marker genes were assessed by qRT‐PCR and normalized to the gene of reference tyrosine 3‐monooxygenase/tryptophan 5‐monooxygenase activation protein zeta (YWHAZ). The evaluation was performed in trophoblasts grown in mono‐cultures (black) and trophoblasts grown in co‐culture with HUVEC (red). Markers were evaluated between days 1 and 7. Values represent mean ± S.E.M, *n* = 4 for mRNA values and *n* = 6 for ELISA. *p < 0.05, **p < 0.01, ***p < 0.001, ****p < 0.0001, trophoblast in co‐culture versus trophoblasts in mono‐culture. ^a^ *p* <0.05 compared to day 4, ^b^ *p* <0.05 compared to day 1.

Firstly, we determined the mRNA expression of hCG as well as the release of hCG protein into the medium from day 1 to day 7. Initially, no significant differences were observed between the two groups. However, on day 7, trophoblast cells in the co‐culture exhibited significantly higher hCG mRNA expression compared to the mono‐culture conditions (Figure 3A). hCG protein release into the medium gradually increased until day 4, followed by a significant elevation on day 5 compared to day 4. These levels remained stable until day 7 (Figure 3B). No significant differences in hCG protein secretion were found between trophoblast cells in mono‐culture and co‐culture (Figure 3B).

Next, we examined the syncytial marker hPL on mRNA and protein levels. We observed an mRNA increase in trophoblast cells growing in co‐culture on days 1, 3 and 7 (Figure 3C). A gradual elevation of hPL protein secretion into the supernatant was apparent in both mono‐ and co‐culture conditions until day 3, with a significant increase on day 4 compared to day 1 (Figure 3D). The secretion of hPL remained stable until day 7 (Figure 3D). Similar to hCG, no significant differences between trophoblast mono‐ and co‐cultures were detected (Figure 3D).

We further investigated the expression of two important syncytial markers involved in cellular fusion, syncytin 1 and syncytin 2 (Figure 3E,F). Syncytin 1 expression was found to be lower in trophoblast cells when grown in co‐culture (Figure 3E), while no significant differences were observed for syncytin 2 expression (Figure 3F).

Furthermore, we analysed the expression of dysferlin, which plays a role in the repair of syncytiotrophoblast membranes. We observed an increase in dysferlin mRNA expression on day 7 in trophoblast cells grown in co‐culture (Figure 3G). Finally, we determined the mRNA levels of PlGF, but no significant differences were found between trophoblast cells grown in mono‐ or co‐cultures (Figure 3H).

### 3.4 Transfer of antipyrine and caffeine in the co‐culture cell model

To further evaluate the biological and biophysical properties of our established in vitro model co‐culture model, we studied the transfer characteristics of different compounds known to cross the placental barrier by diffusion. In these experiments, we used two different compounds: antipyrine, a compound frequently used in human ex vivo placenta perfusion^20^, ^27^ for assessing diffusion properties between the maternal and fetal circulation, and caffeine, as an example of a stimulating agent commonly used during pregnancy. To assess the potentially toxic effects of these compounds on the cells, we additionally performed viability assays utilizing the MTT assay.

**FIGURE 4:**
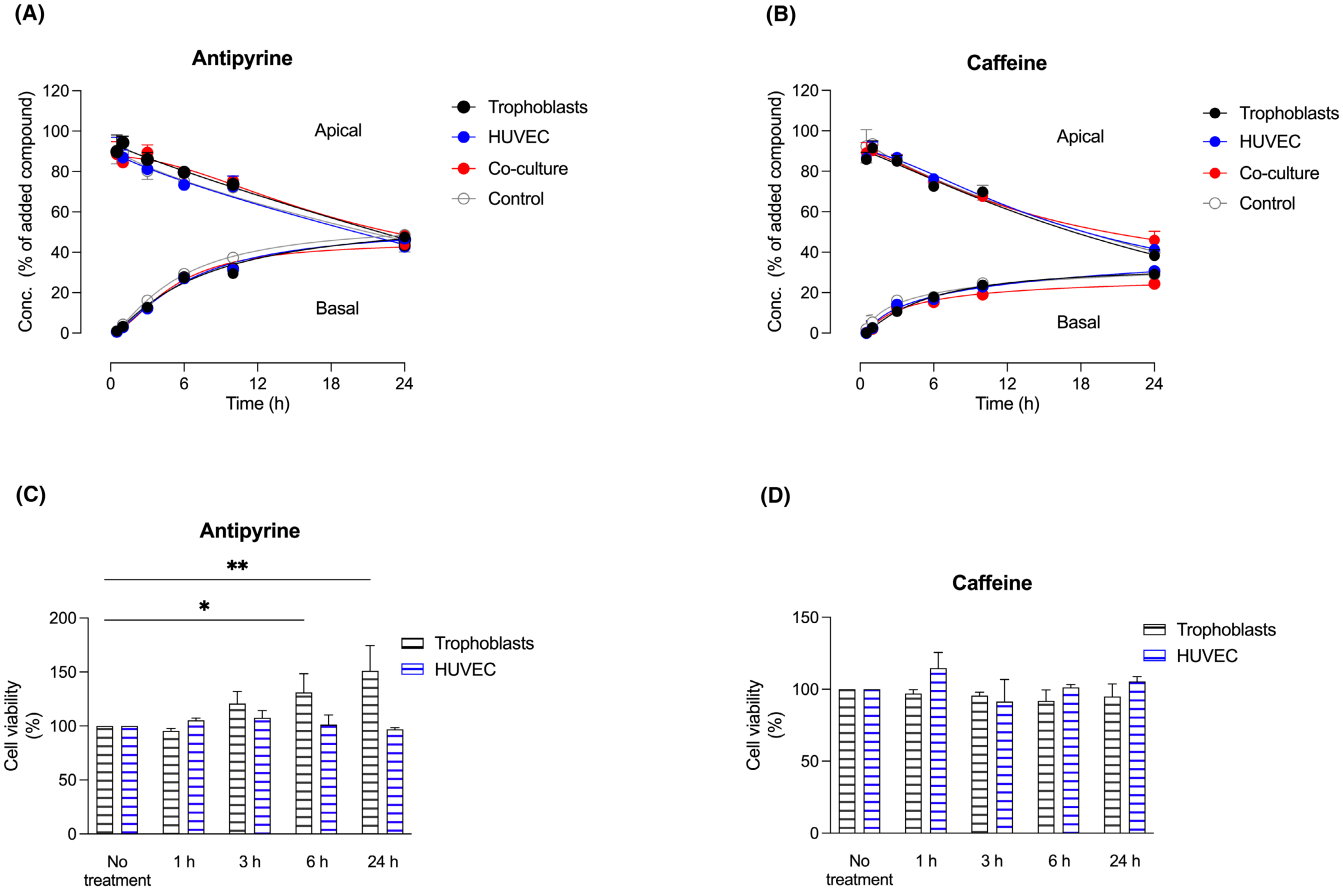
Transfer of antipyrine and caffeine in trophoblast mono‐ and co‐culture models. The apical to basal transfer of (A) 0.43 mM antipyrine, (B) 0.9 mM caffeine, and cell viability of (C) 0.43 mM antipyrine, (D) 0.9 mM caffeine, was assessed in the Transwell^®^ system between 0 and 24 h, in primary trophoblasts grown in mono‐culture (black), primary HUVEC in mono‐culture (blue), primary trophoblasts/primary HUVEC grown in co‐culture (red), and controls (cell‐free Transwell^®^ inserts, grey). Cell viability was assessed using a 3‐(4,5‐dimethylthiazol‐2‐yl)‐2,5‐diphenyltetrazolium bromide (MTT)‐based assay as described in the methods section. Cell viability was evaluated in primary trophoblasts (black) and primary HUVEC (blue) in a 96‐well plate and evaluated between 0 and 24 h. Values are mean ± S.E.M, *p < 0.05, **p < 0.01, *n* = 7 for transfer (trophoblasts, HUVEC and co‐culture, compared to controls) and *n* = 4 for cell viability (trophoblasts and HUVEC compared to no treatment).

Firstly, we assessed the diffusion of antipyrine (0.43 mM), in our cell model over 24 h. In these experiments, we used the same concentration as previously applied in the human placenta perfusion studies. Samples were collected from both the basal and apical compartments of the Transwell^®^ system at various time points. Our results showed an initial increase in the antipyrine concentration in the basal compartment, reaching 26% at 6 h, followed by a saturation phase at 24 h with a maximum basal concentration of 43% (Figure 4A). These findings suggest that antipyrine achieved a diffusional equilibrium between the basal and apical compartments within 24 h.

In Figure 4B results of apical to basal diffusion experiments of caffeine (0.9 mM) are presented. The diffusion reached a plateau after 6 h, with a concentration of 17% in the basal compartment. By 24 h, the concentration increased to 25% (Figure 4B) but did not yet reach an equilibrium between the apical and basal compartments. Importantly, we observed no significant differences between the trophoblasts and HUVEC in mono‐culture and the co‐culture system, nor as compared to the control group (cell‐free Transwell^®^ inserts). The differences in the diffusion rates were not due to potentially toxic effects on the cells since the results of the MTT assay revealed that neither antipyrine (Figure 4C) nor caffeine (Figure 4D) adversely affected cell viability in trophoblasts and HUVEC during 24 h.

### 3.5 LAT1 localization and transport of leucine in the co‐culture cell model

To determine if essential membrane transport proteins are adequately expressed and active in the novel cellular co‐culture model, we performed localization and transport assays in this system. Hereby, we focused on LAT1 since it is known that this amino acid transporter plays a pivotal role in the placenta by transporting essential amino acids such as leucine from the maternal to the fetal circulation.^32^

**FIGURE 5:**
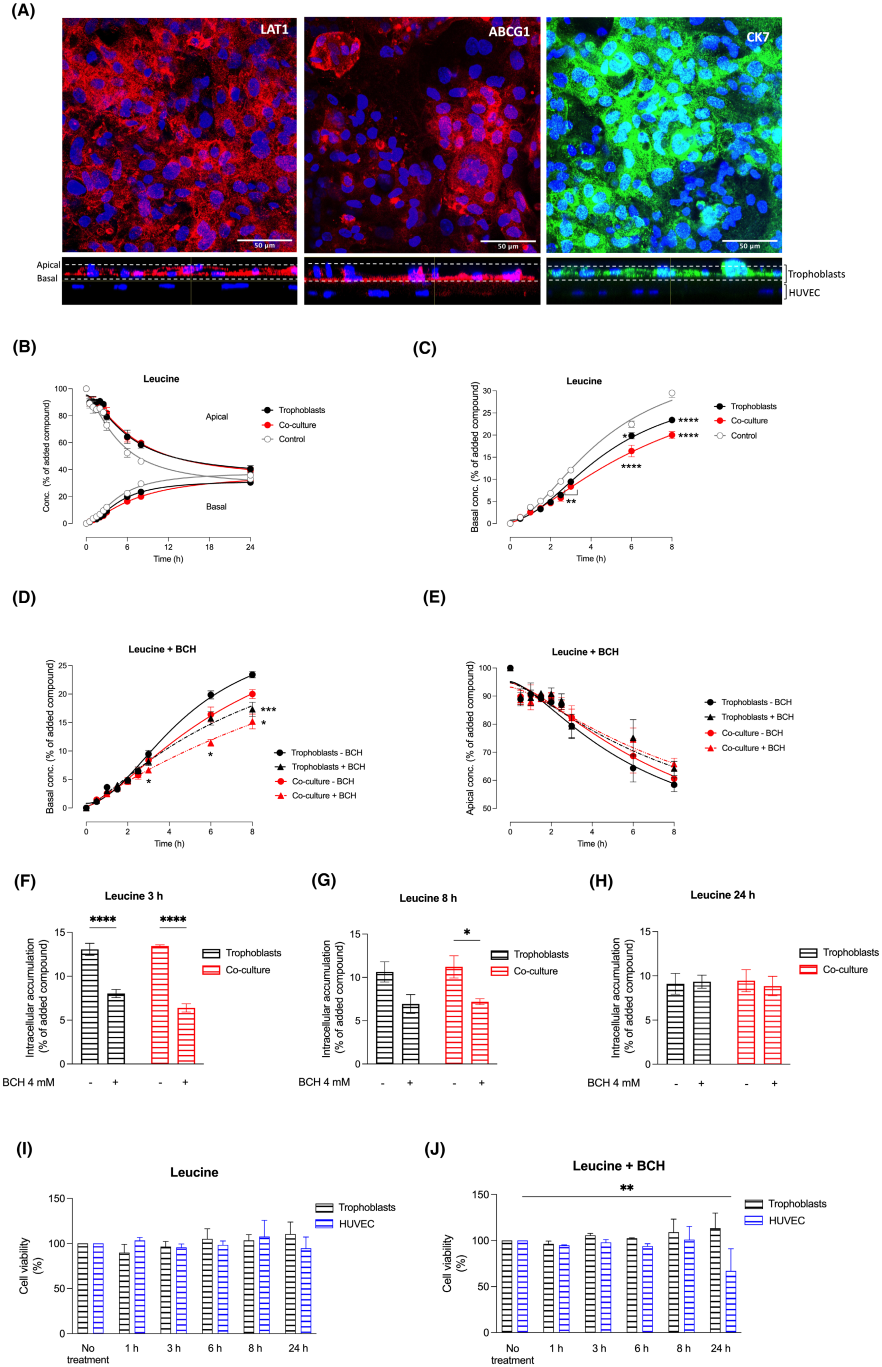
Analysis of L‐type amino acid transporter 1 (LAT1) localization and transport of leucine in the co‐culture cell model. (A) Localization studies performed by confocal microscopy showing the L‐type amino acid transporter 1 (LAT1; left panel), the ATP‐binding cassette transporter G 1 (ABCG1; middle panel), and cytokeratin 7 (CK7, right panel). Staining conditions are explained in detail in the Section 2. (B) Transport of leucine determined in the basal and apical compartment for up to 24 h. (C) Transport of leucine to the basal compartment during 8 h. (D) Transfer of leucine to the basal and (E) disappearance of leucine from the apical compartment in the presence (triangles, dotted lines) or absence (circles, solid lines) of the inhibitor 2‐Amino‐2‐norbornanecarboxylic acid (BCH) in primary trophoblasts (black), co‐cultures of primary trophoblasts and HUVEC (red), and controls (cell‐free Transwell^®^ inserts, grey). (F–H) Intracellular accumulation of [^3^H]‐Leucine in trophoblasts at the end of the experiment, that is, after (F) 3 h, (G) 8 h and (H) 24 h, in the presence (triangles, dotted lines) or absence (circles, solid lines) of BCH. (I + J) Determination of cell viability for primary trophoblasts (black) and HUVEC (blue) after treatment with (I) [^3^H]‐L‐leucine (J) [^3^H]‐L‐leucine + BCH (4 mM, 0–24 h), using a 3‐(4,5‐dimethylthiazol‐2‐yl)‐2,5‐diphenyltetrazolium bromide (MTT)‐based assay as described in the Section 2. Representative images of Z‐stack using orthogonal projections through the Z‐stack, with 40× objective in a Zeiss LSM 710 Confocal microscope Airyscan. *n* = 4–8 per group. *p < 0.05, **p < 0.001, ****p < 0.0001. The cell viability of primary trophoblasts and HUVEC receiving BCH was compared to untreated cells. Similarly, for the quantification of [^3^H]‐L‐leucine, trophoblasts and co‐cultures were compared based on the presence or absence of BCH.

Firstly, we identified the localization of LAT1 in the primary trophoblast cells grown on the Transwell^®^ system in co‐culture with HUVEC. Through z‐stack analysis, we found LAT1 expression both at the apical and basal membrane of the trophoblast (Figure 5A, left panel, red, shown in the Z‐stack images (xz)). To confirm the specificity of apical versus basal localization, we utilized ABCG1 as a marker protein since it was previously demonstrated to be exclusively expressed at the basal membrane of the trophoblast.^22^, ^33^ Indeed, in our trophoblast/HUVEC co‐culture system, ABCG1 showed predominant expression at the basal membrane of trophoblasts (Figure 5A, middle panel). Additionally, CK7, an epithelial marker, was found to be expressed in trophoblast cells (Figure 5A, right panel).

We further investigated the transport of leucine across the Transwell^®^ system over 24 h. Notably, we observed that after approximately 12 h, the concentrations in the apical and basal compartments reached equilibrium (Figure 5B). Figure 5C shows in detail the transport characteristics occurring within the first 8 h and the comparison with cell‐free Transwell^®^ inserts. These results indicate that trophoblasts grown in mono‐culture (black curve) and in co‐culture with HUVEC (red) exhibited decreased leucine transfer to the basal compartment compared to the non‐cell control (grey) (Figure 5C). These results show a reduction in the leucine permeability compared to the cell‐free Transwell^®^ inserts suggesting that the cell layer(s) exert a barrier function.

As expected, the application of the L‐type amino acid inhibitor BCH resulted in a reduction in the transport of leucine to the basal compartment at 3, 6 and 8 h for the co‐culture model, and at 8 h for the trophoblast cells grown in mono‐culture (Figure 5D). Although statistically not significant, there was a trend towards higher leucine concentrations remaining in the apical compartment when BCH was applied, suggesting lower uptake of leucine into the trophoblasts when LAT1 is inhibited (Figure 5E). At 24 h apically and basally no significant differences in mono‐culture and co‐culture in the presence or absence of inhibitor were found (data not shown). Furthermore, inhibition of LAT1 led to decreased intracellular accumulation of leucine after 3 h of incubation with BCH in the trophoblast mono‐ and co‐culture models (Figure 5F). In the co‐culture model, the intracellular leucine concentration was still significantly decreased at 8 h (Figure 5G), but this effect disappeared at 24 h (Figure 5H).

To assess any potentially negative effect of the substrates or inhibitors on cell viability, we incubated the cells with [^3^H]‐leucine (Figure 5I) and [^3^H]‐leucine in combination with BCH (Figure 5J). There were no differences in cell viability after up to 24 h of incubation in trophoblast cells. In HUVEC, however, cell viability decreased to 60% after 24‐h exposure to BCH.

### 3.6 P‐glycoprotein localization and transport of Rhodamine 123 in the co‐culture cell model

**FIGURE 6:**
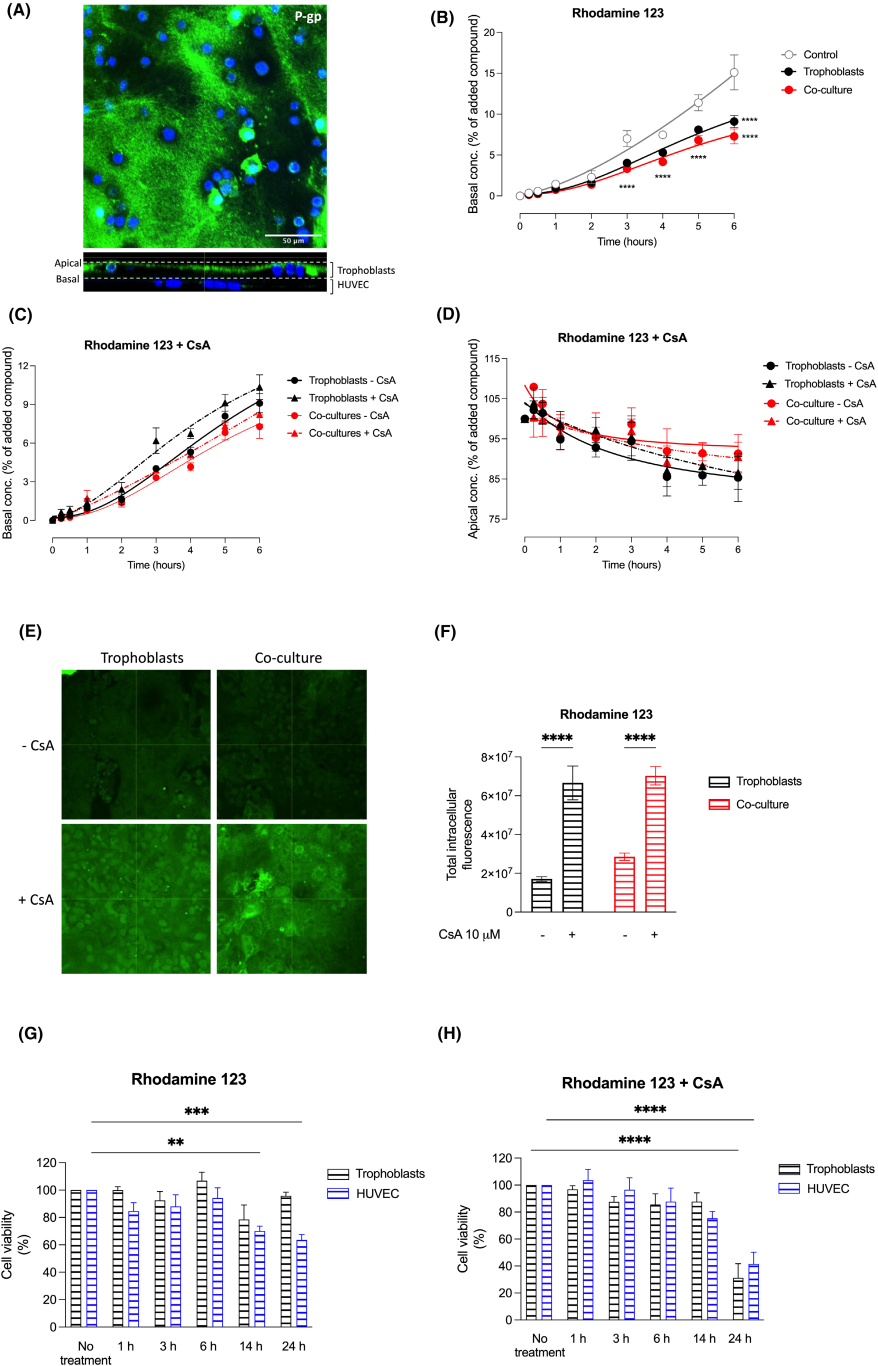
Analysis of P‐glycoprotein (P‐gp) localization and transport of Rhodamine 123 in the co‐culture cell model. (A) Localization of P‐glycoprotein (P‐gp) in primary trophoblasts grown in co‐culture with HUVEC in the Transwell^®^ system. The immunofluorescence image shows the merge of DAPI and the corresponding staining with the P‐gp antibody. A representative image of the Z‐stack using orthogonal projections through the Z‐stack was taken with a 40× objective in a Zeiss LSM 710 Confocal microscope Airyscan. *n* = 3–4 per group. Staining conditions are explained in detail in the Section 2. (B) Transport of Rhodamine 123 to the basal compartment during 6 h. (C) Transport of Rhodamine 123 to the basal compartment and (D) disappearance of Rhodamine 123 from the apical compartment in the presence (triangles, dotted lines) or absence (circles, solid lines) of the inhibitor Cyclosporine A (CsA) in primary trophoblasts (black), co‐cultures of primary trophoblasts and HUVEC (red), and controls (cell‐free Transwell^®^ inserts, grey). (E) Intracellular accumulation of Rhodamine 123 at the end of the experiment (6 h), in trophoblast mono‐ and co‐cultures, in the presence or absence of CsA. Representative images were taken from a total of three analysed Transwell^®^ inserts per group. (F) Quantification of the intracellular Rhodamine 123‐based fluorescence. (G + H) Determination of cell viability after application of (G) Rhodamine 123 (H) Rhodamine 123 + CsA (10 μM, 0–6 h), using a 3‐(4,5‐dimethylthiazol‐2‐yl)‐2,5‐diphenyltetrazolium bromide (MTT)‐based assay as described in the Section 2. The cell viability was evaluated in primary trophoblasts (black) and HUVEC (blue), in a 96‐well plate and evaluated between 0 and 24 h. ***p < 0.001, ****p < 0.0001. The cell viability of primary trophoblasts and HUVEC receiving CsA was compared to untreated cells. Similarly, for the quantification of Rhodamine 123, trophoblasts and co‐cultures were compared based on the presence or absence of CsA.

To determine the effectiveness of our model for evaluating drug transport processes across the placenta, we studied the localization and functional activity of P‐gp, one of the most important efflux transporters for various drugs. First, we determined by confocal microscopy its localization in primary trophoblasts grown on the Transwell^®^ system in co‐culture with HUVEC. We found that P‐gp is predominantly present at the apical membrane of trophoblast cells (Figure 6A).

Next, we evaluated the transport of the P‐gp substrate Rhodamine 123 from the apical to the basal compartment. We detected significantly lower transfer rates to the basal compartment when trophoblasts were grown on the Transwell inserts in mono‐ (black) or co‐culture with HUVEC (red) compared to cell‐free control inserts (grey) (Figure 6B). Furthermore, we assessed the effect of the P‐gp inhibitor CsA on the transport characteristics of Rhodamine 123. Four different concentrations of Rhodamine 123 were analysed in primary trophoblasts and tested in the presence of CsA. These experiments revealed that efflux of 10 μM Rhodamine 123 was adequately inhibited by 10 μM CsA (Figure S2). In the Transwell^®^ experiments, however, no significant differences were found after application of CsA concerning the transfer rates to the basal compartment (Figure 6C) and the remaining Rhodamine 123 concentration at the apical side (Figure 6D). In contrast, when determining the intracellular Rhodamine 123‐based fluorescence by confocal microscopy, we observed increased accumulation of Rhodamine 123 in trophoblast cells grown in mono‐ and co‐cultures (Figure 6E) when the inhibitor CsA was applied. Quantification of the intracellular fluorescent signal revealed approximately three times higher intracellular Rhodamine 123 concentrations in trophoblasts and co‐cultures after inhibition of P‐gp with CsA (Figure 6F). Subsequently, we assessed cell viability using Rhodamine 123 (Figure 6G), and Rhodamine 123 combined with the P‐gp inhibitor, CsA (Figure 6H). We found no significant differences in cell viability up to 6 h in both trophoblasts and HUVEC. However, after 24 h, we observed a decrease in cell viability of approximately 40% in HUVEC after incubation with Rhodamine (Figure 6G), and approximately 60% in trophoblasts and HUVEC after incubation with Rhodamine 123 plus CsA (Figure 6H). Therefore, the transport experiments with Rhodamine 123 were limited to 6 h.

## 4 DISCUSSION

The development of in vitro models that accurately mimic the complexity and functionality of the placental barrier is essential not only for studying (patho)physiological processes occurring at the maternal‐fetal interface but also for monitoring drug transport across the human placenta allowing the prediction of fetal exposure to drugs. In this study, we aimed to establish and validate a co‐culture cell model consisting of primary trophoblast cells and primary HUVEC grown simultaneously on either side of Transwell^®^ inserts. In this co‐culture model—which shall more realistically resemble the placental barrier—we investigated monolayer formation and tightness, syncytialization markers, cell viability and hormone release. Moreover, we evaluated physiologically important aspects such as transplacental diffusion and carrier‐mediated transport processes of compounds which are relevant for maternal and/or fetal wellbeing during pregnancy. By monitoring and evaluating several crucial parameters that are linked to the role and function that the human placenta has in vivo, we gained valuable insights into the functionality of this new co‐culture model.

Given the complexity of establishing a monolayer formation in primary trophoblasts and synchronizing the cellular growth of both cell types, various experiments were performed to monitor the development of a tight monolayer. These experiments aimed to find adequate conditions to ensure successful monolayer formation of trophoblasts and HUVEC and to prevent leakage of media between the apical and basal compartments. In this context, we used the cellZscope^®^ device which monitors TEER and Ccl, providing insights into the structure and function of the cell layer.^34^, ^35^ These measurements captured the resistance contributed by tight junction proteins between cells, as well as the resistance within the cells. Using this experimental setup, we found that TEER values were increased after day 1 in the trophoblast/HUVEC co‐cultures and were higher compared to trophoblasts and HUVEC grown in mono‐culture. These results indicate that the formation of a monolayer including tight cell junctions started already 1 day post seeding and continued to develop until approximately day 3–4. Thereafter, the TEER values reached a plateau and remained stable throughout the entire time of the experiments. In addition to measuring TEER values, the cellZscope^®^ system also records the cell layer's capacitance (Ccl), which is an indicator of the membrane surface area. The decrease in capacitance indicates improved cell–cell interactions and reduced paracellular diffusion of ions, resulting from tight junction formation. Ccl values ranging from 0.5 to 5.0 μF/cm^2^ signify cell confluency and serve to validate TEER values.^36^, ^37^ The fact that we observed lower Ccl values in the co‐culture system already at day 2 could indicate beneficial growth conditions leading to a better cell confluency at the early stages of monolayer formation.

In the next step, we evaluated the paracellular permeability of the monolayer(s) by measuring the transfer of LY and calculating the Papp. For the different cell preparations (trophoblasts, HUVEC and co‐cultures), the mean Papp values for LY were significantly lower from day 3 onwards compared to cell‐free control Transwell^®^ inserts. The Papp values ranged from 1.6 to 3.3 × 10^6^ cm/s which is comparable or even lower than the ones reported for an intact Caco‐2 monolayer (2.5–5.0 × 10^6^ cm/s).^38^ In the context of monolayer tightness, we studied besides LY (457 Da) also the higher molecular weight compound inulin (5200 Da). We observed that the transfer of inulin to the basal compartment was approximately 0.28% after 2 h for all three cell types (trophoblasts, HUVECs and co‐culture). However, for the larger substrate inulin, which is neither transported via a specific mechanism nor metabolized,^39^ it took longer to cross the cell layers and reach the basal compartment. These findings further confirmed the presence of a tight monolayer, since it has been described that a leakage <1%/h is the maximum acceptable leakage rate^40^ to ensure the integrity and effective barrier function of the cells. The findings in our co‐culture model revealed transfer percentages of 2%, 3.6% and 10% after 6, 10 and 24 h, respectively, aligning with the recommended criteria.

The formation of a monolayer was observed both in mono‐ and co‐culture conditions, as demonstrated by immunofluorescence staining with the epithelial marker CK7 and the endothelial marker CD31. Interestingly, trophoblast cells in the co‐culture condition exhibited faster growth, with monolayer formation observed as early as on day 3 compared to trophoblasts in mono‐culture where this occurred approximately by day 5. This differential growth pattern suggests potential intercellular influences, possibly mediated by secreted proteins that promote cellular growth. In other cell models, it has been previously demonstrated that co‐culturing 3D JEG‐3 cell spheres with human brain microvascular endothelial cells led to significantly elevated expression levels of syncytialization markers, such as hCG and hPL.^41^ Our results suggest a possible similar interplay between trophoblasts and endothelial cells, enhancing selected syncytialization markers.

In the context of syncytialization, we also assessed the mRNA levels of six syncytial markers in primary trophoblast mono‐cultures and trophoblast co‐cultures at four different time points. Our results showed increased expression of hCG and dysferlin at day 7, and hPL at days 1, 3 and 7 in trophoblasts grown in co‐culture compared to mono‐culture conditions. Conversely, higher expression of syncytin 1 was observed in trophoblast mono‐cultures. In this regard, our study contributes to the comprehension of trophoblast marker expression during the formation of monolayers and syncytialization. Notably, the mRNA expression patterns of hCG and hPL, which are important and frequently used markers for syncytialization,^21^ underline the importance of utilizing co‐culture models for studying placental transport in a more physiological context. The increased expression of dysferlin suggests its utility as a reliable marker of syncytialization due to its role in plasma membrane repair.^42^, ^43^, ^44^ Additionally, the expression pattern of hPlGF highlights its relevance in trophoblast development.^45^ On the other side, our observations regarding syncytin 1 and 2 contribute to the ongoing debate on their functions in trophoblast fusion.^46^

Additionally, we analysed hCG and hPL protein secretion into the cell culture media of trophoblast mono‐cultures and trophoblast co‐cultures as parameters of cell differentiation. The observed secretion patterns verified the progress of trophoblast differentiation. The stable secretion of hCG and hPL once the monolayer was formed (day 5) suggests a similar differentiation progress in both groups. Regarding the hormone release from trophoblast mono‐ and co‐cultures, no differences were observed between the two groups despite of partially increased mRNA expression levels of hCG and hPL in the co‐culture system. These data suggest that once the monolayer is formed and the syncytialization process is completed, the primary trophoblasts reach and maintain their maximal secretion capacity.

After having verified the formation of tight and functional monolayers in our co‐culture system we tested the new cell model for the transfer capacity of different compounds known to cross the placental barrier either by diffusion or carrier‐mediated transport processes. To assay the diffusion characteristics, we used both antipyrine, a frequently utilized reference compound in human placental perfusion experiments to track passive diffusion^47^, ^48^ and caffeine, a stimulating agent commonly used during pregnancy. Both molecules are known to freely diffuse across the placental barrier.^28^, ^48^ For comparative purposes, we utilized the same concentration of antipyrine, as commonly applied in human placental perfusion studies.^20^, ^27^, ^47^, ^48^ The concentration of caffeine was previously evaluated in a series of in vitro studies where it ranged between 0.25 and 1.55 mM.^27^, ^28^ For both compounds (antipyrine and caffeine), we found similar permeability properties between trophoblasts, HUVECs and co‐cultures.

Since the initial tests regarding monolayer formation, syncytialization, hormone release and diffusional transfer capacity yielded positive and promising results, we finally analysed the functional properties of the new co‐culture model. Herein, we focused on two physiologically important roles of the placenta, namely nutrient transport and protection of the fetus against drug exposure. In this context, we first determined if essential membrane transport proteins are adequately expressed and active in the novel cellular co‐culture model by performing localization and transport assays in this system. Hereby, we focused on LAT1 since it is known that this amino acid transporter plays a pivotal role in the placenta by transporting essential amino acids from the maternal to the fetal circulation.^32^ Leucine, one of the substrates of LAT1, is an essential amino acid that needs to be taken up by the diet and plays a crucial role in supporting cellular functions during pregnancy.^49^ It is vital for synthesizing proteins, which is essential for the growth and development of the fetus.^50^, ^51^ In addition, leucine contributes to regulating blood sugar levels and serves as a significant energy source for both the mother and the developing fetus.^52^ It has been previously reported that LAT1 is present in HUVEC.^53^ Regarding its localization in trophoblasts, the current literature presents conflicting information: some reports indicate expression solely at the apical membrane of the trophoblast,^54^, ^55^, ^56^ while others suggest expression in both the apical and basal membrane.^57^, ^58^ Our results obtained by confocal microscopy suggested the presence of LAT1 at both the apical and basal membrane of syncytialized primary trophoblast cells. Although the function of LAT1 has been primarily associated with amino acid uptake at the apical membrane, the observed localization at the basal membrane of polarized trophoblasts may suggest also a potential role of LAT1 in the release of amino acids to the fetal blood. To validate the localization assay performed by confocal microscopy, we employed two control measures: Firstly, we tested the expression of ABCG1, known to be expressed at the basal membrane of the trophoblast,^22^, ^59^ and indeed we found this transporter exclusively localized at the basal membrane. The second control, CK7, displayed—as expected^60^—a ubiquitous presence across all trophoblasts.

Within the performed transport experiments, we observed that at 24 h the equilibrium of L‐leucine transport was delayed in trophoblasts and co‐culture compared to cell‐free control inserts. This indicates the presence of transporter‐mediated transfer processes through the cell layers, rather than diffusion across the Transwell^®^ membrane. Subsequently, we examined leucine transport using BCH, an inhibitor of L‐system amino acid transporters, which previously had been shown to inhibit leucine uptake in trophoblast cells^56^ and LAT1 in HUVEC.^61^ We observed a marked decrease in leucine transport across the co‐culture in the presence of BCH after 3, 6 and 8 h. At the 8 h time point, a significant (approximately 25%) reduction of transfer was detected in trophoblast cells in both mono‐culture and co‐culture. The inhibition of L‐type system amino acid transporters by BCH affected mostly amino acid transport to the basal compartment and intracellular concentrations in both the co‐culture and trophoblast mono‐cultures.

Finally, we aimed to determine the effectiveness and applicability of our model for evaluating drug transport across the placental barrier. Hence, we studied the localization and functional activity of P‐gp, one of the most important efflux transporters for various drugs, potentially safeguarding the fetus from xenobiotics.^62^, ^63^, ^64^ As expected and indicated in the literature,^62^ we detected P‐gp expression at the apical membrane of primary syncytialized trophoblasts. It has been previously reported that P‐gp expression in the trophoblast cell line BeWo is very low,^65^ underscoring the importance of employing primary trophoblast cells for drug transport studies in a physiologically relevant context. HUVEC exhibits very low or negligible P‐gp expression,^66^ and endothelial cells of placental fetal capillaries were shown to lack it completely.^67^

We assessed the transport of Rhodamine 123 and found reduced transfer to the basal compartment in trophoblasts (mono‐ and co‐cultures) compared to cell‐free control inserts. Rhodamine 123 is a substrate commonly used to study the function of the P‐gp transporter^68^ and serves as a fluorescent indicator capable of entering cells. P‐gp, being an efflux pump, actively exports a variety of compounds—among them Rhodamine 123^69^—out of the cell. In our co‐culture experiments, only 7% of the applied Rhodamine 123 was detected in the basal compartment compared to 15% in the controls (cell‐free Transwell^®^ inserts). Rhodamine 123 transport in the presence or absence of CsA—as assessed by measuring fluorescence in the basal or apical compartments—did not show significant differences, although there was a trend towards increased fluorescence in the basal compartment when the inhibitor was present. However, subsequent evaluation of the intracellular fluorescence showed increased Rhodamine 123 levels after inhibition of P‐gp, suggesting an accumulation of the substrate within the cells. Generally, it is well accepted that CsA acts by altering the protein conformation of P‐gp, impeding its active efflux activity.^70^ This can lead to increased intracellular concentrations of drugs as also detected in our cell model. Evaluation of cell viability post exposure to Rhodamine 123 and/or the inhibitor CsA showed no adverse effects during 6 h, but increased cytotoxicity in trophoblasts when CsA was applied for 24 h. We therefore opted for a 6 h duration of the Rhodamine 123 transfer experiments. This chosen time course, however, may have prevented the occurrence of significant differences since diffusion processes of intracellular Rhodamine 123 through the basal trophoblast membrane and the HUVEC monolayer to the basal compartment may require more time.

In conclusion, our newly established co‐culture cell model recapitulates major physiological features and functions of the naturally occurring human placental barrier. Thus, it provides valuable insights into monolayer formation, syncytialization processes, hormone release and the transport of different physiologically or pathophysiologically relevant compounds. These findings contribute to our understanding of placental function in general and will advance our knowledge of placental barrier function and drug transport processes across the human placenta. Future research in this area could benefit from a more comprehensive exploration of a broader range of markers and transporters to gain a deeper understanding of the mechanisms underlying nutrient and drug transport to the developing fetus. Additionally, to improve the model's physiological relevance even further, future directions could involve the incorporation of a flow component into the co‐culture system intended to mimic maternal and/or fetal blood circulation.

## AUTHOR CONTRIBUTIONS

**Barbara Fuenzalida:** Conceptualization (equal); data curation (lead); formal analysis (lead); methodology (lead); writing – original draft (lead). **Virginia Basler:** Data curation (supporting); formal analysis (supporting); methodology (supporting); writing – review and editing (supporting). **Nadja Koechli:** Data curation (supporting); formal analysis (supporting); methodology (supporting). **Nan Yi:** Data curation (supporting); formal analysis (supporting); methodology (supporting); writing – review and editing (supporting). **Frantisek Staud:** Conceptualization (supporting); data curation (supporting); formal analysis (supporting); methodology (equal); supervision (supporting); validation (supporting); writing – review and editing (supporting). **Christiane Albrecht:** Conceptualization (equal); funding acquisition (lead); methodology (supporting); project administration (lead); resources (lead); supervision (lead); validation (equal); writing – review and editing (lead).

## FUNDING INFORMATION

This study was supported by the Swiss 3R Competence Centre (3RCC; grant no OC‐2019‐019, CA) and the Swiss National Science Foundation (grant no. 310030_197408; CA).

## CONFLICT OF INTEREST STATEMENT

The authors declare no conflict of interest.

## Supporting information


Figures S1–S2.

## Data Availability

The data and material that support the findings of this study are available upon reasonable request from the corresponding author (christiane.albrecht@unibe.ch).
