# Supplementary material for: Modelling the maternal‐fetal interface: An in vitro approach to investigate nutrient and drug transport across the human placenta
Source: J Cell Mol Med. 2024 Oct 18;28(20):e70151. doi: 10.1111/jcmm.70151 (PMC11487339; doi:10.1111/jcmm.70151)
Supplement: Supplementary file 1 — Figures S1–S2. [file JCMM-28-e70151-s001.docx]

**Modeling the Maternal-Fetal Interface: An In Vitro Approach to Investigate Nutrient and Drug Transport Across the Human Placenta**

Barbara Fuenzalida^1^, Virginia Basler^1^, Nadja Koechli^1^, Nan Yi^1^, Frantisek Staud^2^, Christiane Albrecht^1^

^1^ Institute of Biochemistry and Molecular Medicine, Faculty of Medicine, University of Bern, 3012 Bern, Switzerland.

^2^ Department of Pharmacology and Toxicology, Faculty of Pharmacy in Hradec Kralove, Charles University, Hradec Kralove, Czech Republic.


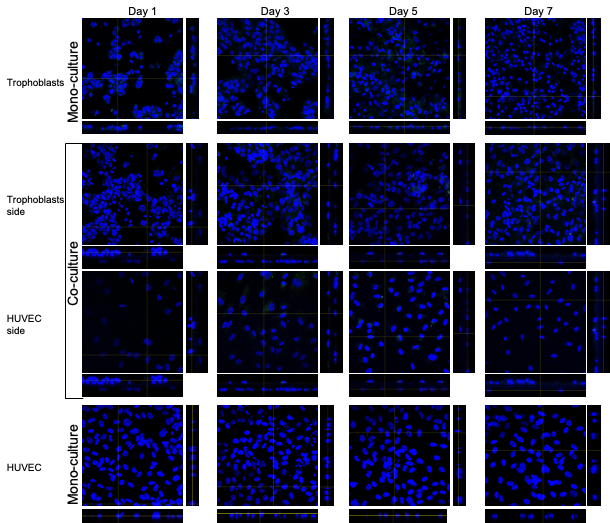


**Figure S1**. Negative control for immunofluorescence in Transwell^®^ inserts between days 1 and 7, shown in figures 2, 5A, and 6A. The secondary antibodies were Alexa fluor 488-conjugated goat anti-mouse (for trophoblast cells) and Alexa Fluor 568-conjugated goat anti-rabbit (for HUVEC).


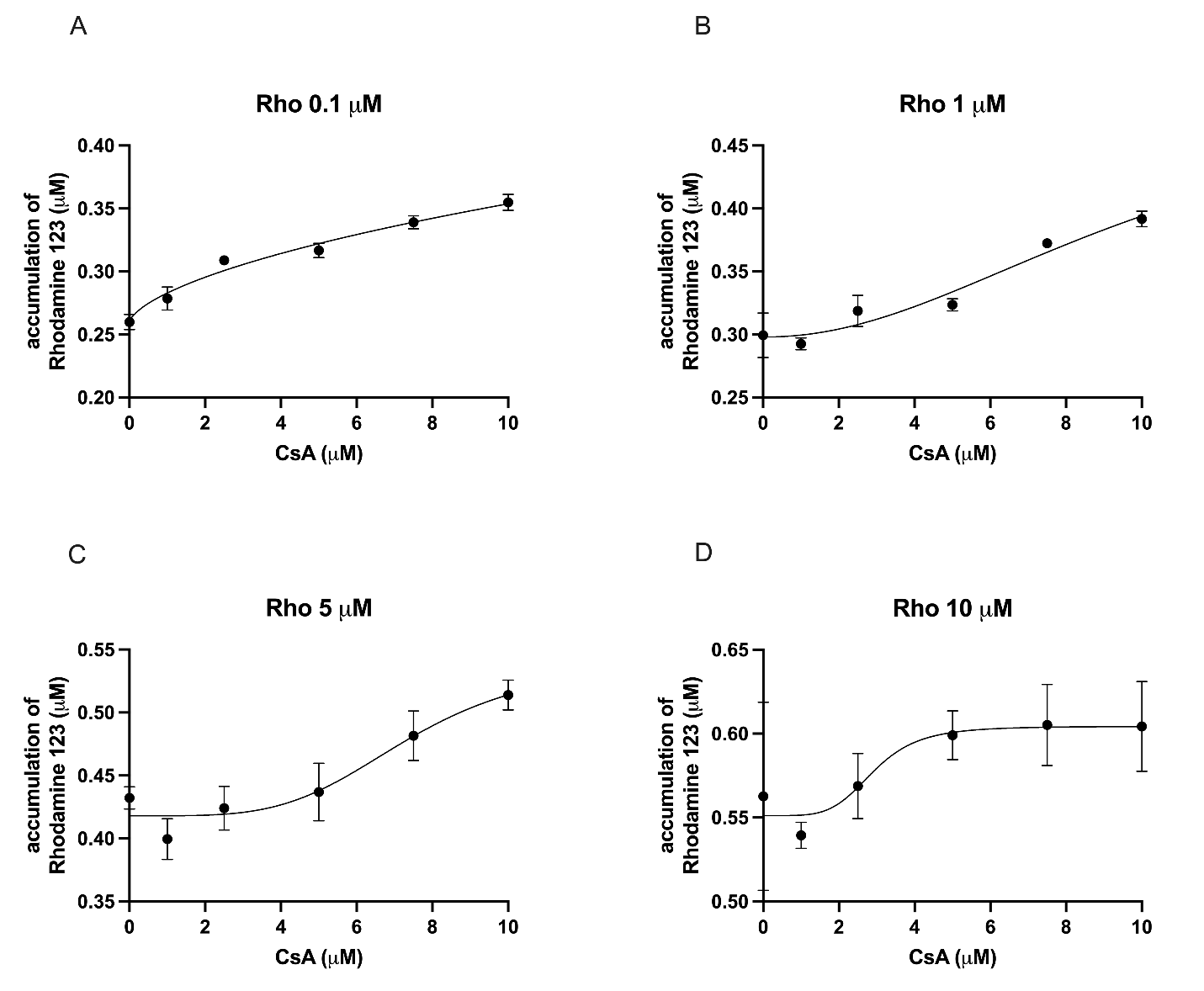


**Figure S2**. Inhibition of Rhodamine 123 efflux in primary trophoblast cells by cyclosporine A (CsA). Trophoblasts were seeded at a density of 1x10^5^ on a 96 well plate. After 48 h Rhodamine 123 (Rho) at a concentration of (A) 0.1 µM, (B) 1 µM, (C) 5 µM and (D) 10 µM was applied. Intracellular fluorescence was evaluated at 3 h in the presence of 1 - 10 µM CsA using a fluorescence microplate reader at Ex/Em = 500/525 nm. Values were corrected for the protein concentrations as determined by bicinchoninic acid (BCA) assay. n=3.
